# Microvascular Osteocutaneous Free-Flap Reconstruction of Complex Maxillary Defects: A Two-Year Single-Center Experience

**DOI:** 10.7759/cureus.113987

**Published:** 2026-08-05

**Authors:** Mainak Mallik, Akanksha Rajpoot, Sunil Rout, Priyanka Pant

**Affiliations:** 1 Burns and Plastic Surgery, All India Institute of Medical Sciences, Kalyani, Kalyani, IND; 2 Burns and Plastic Surgery, Father Muller Medical College Hospital, Mangalore, IND; 3 Burns and Plastic Surgery, Kalinga Institute of Medical Sciences (KIMS), Bhubaneswar, IND; 4 Burns and Plastic Surgery, Pan Singh Pimoli Multi Super Speciality Hospital and Research Centre, Haldwani, IND

**Keywords:** brown’s classification, fibula flap, maxillary reconstruction, microvascular flap, mucormycosis, radial forearm flap

## Abstract

Maxillary defects resulting from oncologic resection, severe trauma, or invasive infections such as mucormycosis present significant reconstructive challenges due to the need to restore midfacial support, oral competence, speech, mastication, and facial aesthetics. Microvascular osteocutaneous free flaps have expanded reconstructive options by providing vascularized bone and soft tissue for simultaneous restoration of structural support and intraoral lining. This report describes our reconstructive experience in three patients who underwent microvascular reconstruction of complex maxillary defects at our institution over a two-year period. Etiologies included invasive mucormycosis in one patient and oncologic resection in two patients. Defects were classified according to the revised Brown classification, with one type IIIb defect involving the maxilla and orbital floor and two type V defects involving the maxilla and adjacent midfacial structures. Reconstruction was performed using free fibula osteocutaneous flaps in two patients and a radial forearm osteocutaneous flap in one patient, selected according to defect characteristics and tissue requirements. Bony segments were contoured to recreate the maxillary framework, while skin paddles were used for intraoral lining and oral-nasal separation. All flaps survived completely, with satisfactory healing, restoration of midfacial contour and support, preservation of oral competence and speech, and acceptable facial symmetry. Minor secondary contouring procedures were required in selected patients. No flap failures, major complications, or significant donor-site morbidity were observed. This case series demonstrates the application of osteocutaneous free flaps in the reconstruction of complex maxillary defects and highlights important technical considerations and the use of an algorithmic approach in flap selection based on the revised Brown classification.

## Introduction

Complex maxillary defects following oncologic resection, craniofacial trauma, or invasive infections such as post-COVID mucormycosis present significant reconstructive challenges in oral and maxillofacial surgery. Owing to the maxilla's central role in maintaining facial contour, orbital support, dental occlusion, mastication, speech, and separation of the oral and nasal cavities, successful reconstruction requires restoration of the bony framework, mucosal lining, and soft tissue to achieve satisfactory functional and aesthetic outcomes [1-4].

Brown's revised classification system (2010) provides a structured anatomical framework for categorizing maxillary defects and guiding reconstructive planning [5]. High-grade defects, including types IIIb and V, often involve complex three-dimensional tissue loss requiring composite tissue reconstruction. Although prosthetic obturation was historically the primary treatment modality, its limitations in restoring extensive defects have led to increasing use of microvascular osteocutaneous free flaps. Among these, the fibula osteocutaneous flap remains the workhorse for extensive maxillary reconstruction because of its reliable vascularity, ample cortical bone, long pedicle, and suitability for dental rehabilitation [6,7]. The radial forearm osteocutaneous flap, while providing limited bone stock, offers thin, pliable soft tissue and is particularly useful in selected defects where internal lining and cavity obliteration are the primary reconstructive goals [8].

We present three cases of complex maxillary reconstruction using microvascular osteocutaneous free flaps to illustrate defect-based flap selection, technical considerations, and reconstructive outcomes. The study was conducted in accordance with the Declaration of Helsinki, and informed written consent was obtained from all patients.

## Case presentation

Case 1: Brown type IIIb defect

**Figure 1:**
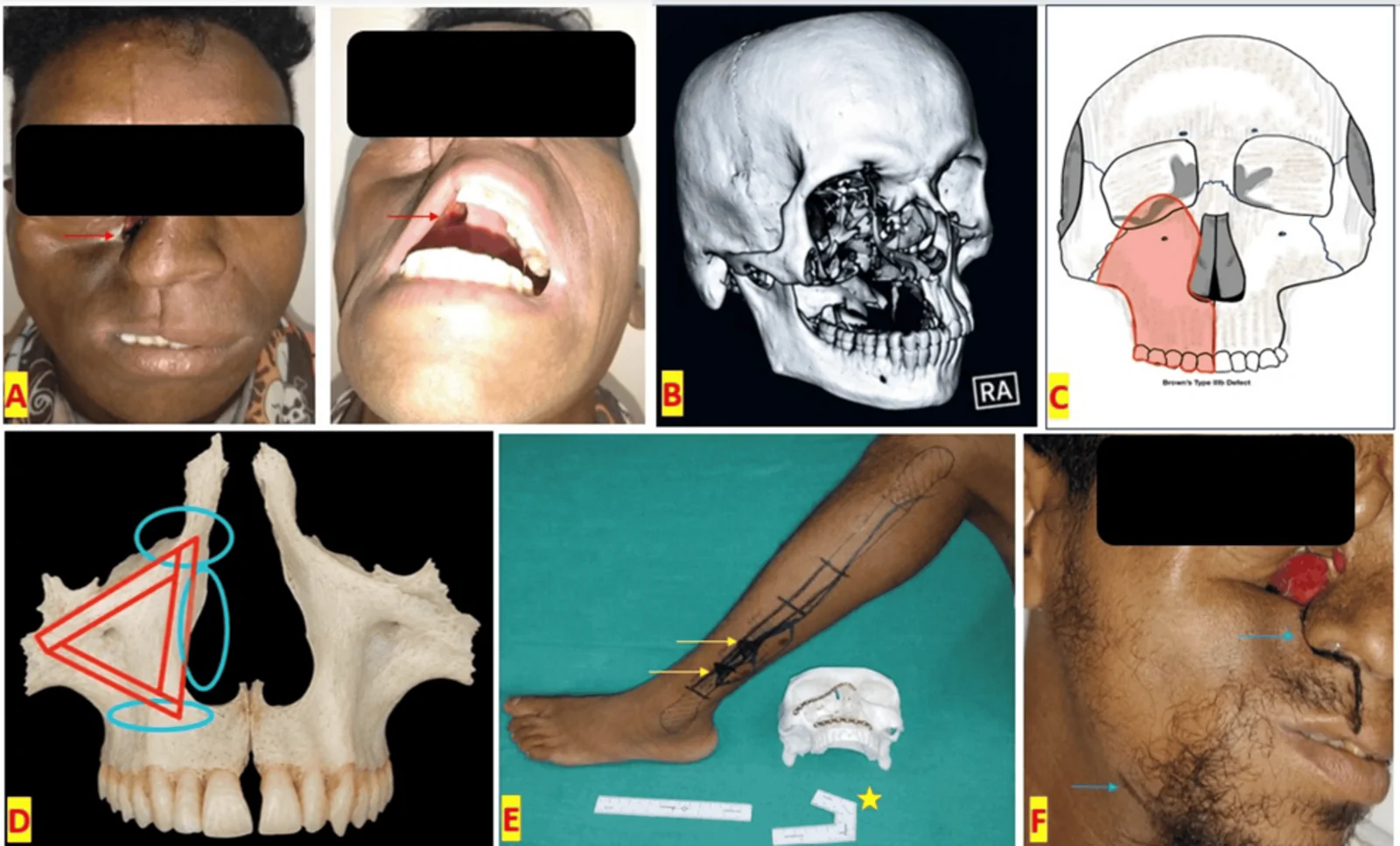
Images of patient 1. (A) A 35-year-old male with a post-surgical maxillary defect (red arrow) with an oroantral fistula and a failed ipsilateral paramedian forehead flap. (B) Non-contrast computed tomography (NCCT) with 3D reconstruction showing a right orbito-maxillary defect involving the palate. (C) Illustration showing the defect as per revised Brown classification – type IIIb (Digital drawing created by the corresponding author using Notability, Ginger Labs, Inc., San Francisco, CA). (D) Surgical planning for reconstruction. Red solid lines depict reconstruction of the infraorbital rim, nasomaxillary buttress, and zygomaticomaxillary buttress. Blue solid lines show the placement of skin flaps as lining for the nasal and oral cavity. (E) Reconstruction plan – Planning of contralateral fibula osteocutaneous free flap with templates for future osteotomies and fixation with 2 mm reconstruction plates (yellow solid arrow - sites of osteotomy; yellow star - measuring scale template showing future shape after osteotomy). (F) Surgical approach – Weber Ferguson’s incision and submandibular incision for donor vessel dissection (blue arrows).

**Figure 2:**
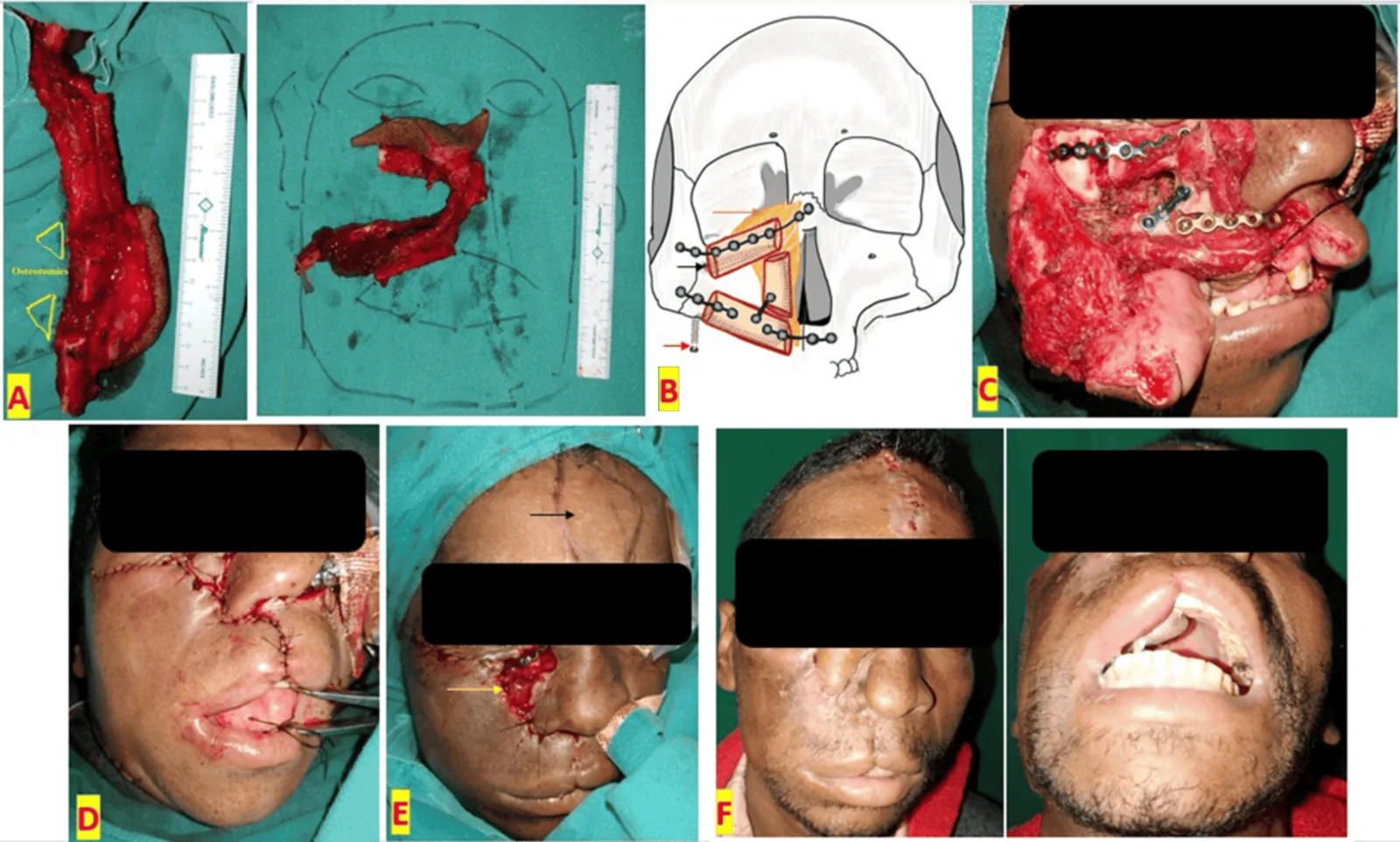
Patient 1. (A) Fibula osteocutaneous free flap showing level of vertical osteotomies. (B) Illustrations showing flap orientation and planned inset of flap (orange arrow – skin paddle as inner mucosal lining; black arrow – bone fixation; red arrow – pedicle orientation to right side of the neck) (Digital drawing created by the corresponding author using Notability). (C) Flap inset to the defect and fixation with 2 mm reconstruction miniplates and screws. (D) Final closure. (E) Small area of flap necrosis at nasomaxillary region (yellow arrow). Debridement followed by resurfacing with contralateral paramedian forehead flap (black arrow). (F) Follow-up at one month with a well-settled fibula and paramedian forehead flap.

A 35-year-old male presented with a post-oncologic maxillectomy defect involving the orbital floor, anterior maxillary wall, and hard palate following resection of squamous cell carcinoma of the right maxilla. The patient had previously undergone reconstruction with an ipsilateral paramedian forehead flap, which subsequently failed, resulting in a persistent oronasal fistula and significant midfacial contour deformity. Reconstruction with a free fibula osteocutaneous flap was planned. Intraoperatively, a 10 cm segment of fibula with a single skin paddle was harvested. The bony component was contoured and inset to reconstruct the orbital floor and anterior maxillary buttress, while the skin paddle was positioned intraorally to line the palatal defect. Microvascular anastomosis was performed using the facial artery and vein as recipient vessels. Free flap monitoring was done hourly on postoperative day 0 and 1, followed by four-hourly on days 2 and 3. The postoperative course was largely uneventful except for a small area of marginal flap necrosis in the right nasomaxillary region, which was subsequently managed with debridement and resurfacing using a contralateral paramedian forehead flap. At three months, the patient underwent a minor debulking procedure to reduce the intraoral flap. Satisfactory speech, swallowing, and maintenance of normal orbital position were achieved postoperatively, as observed at six-month follow-up (Figures 1, 2).

Case 2: Brown type V defect

**Figure 3:**
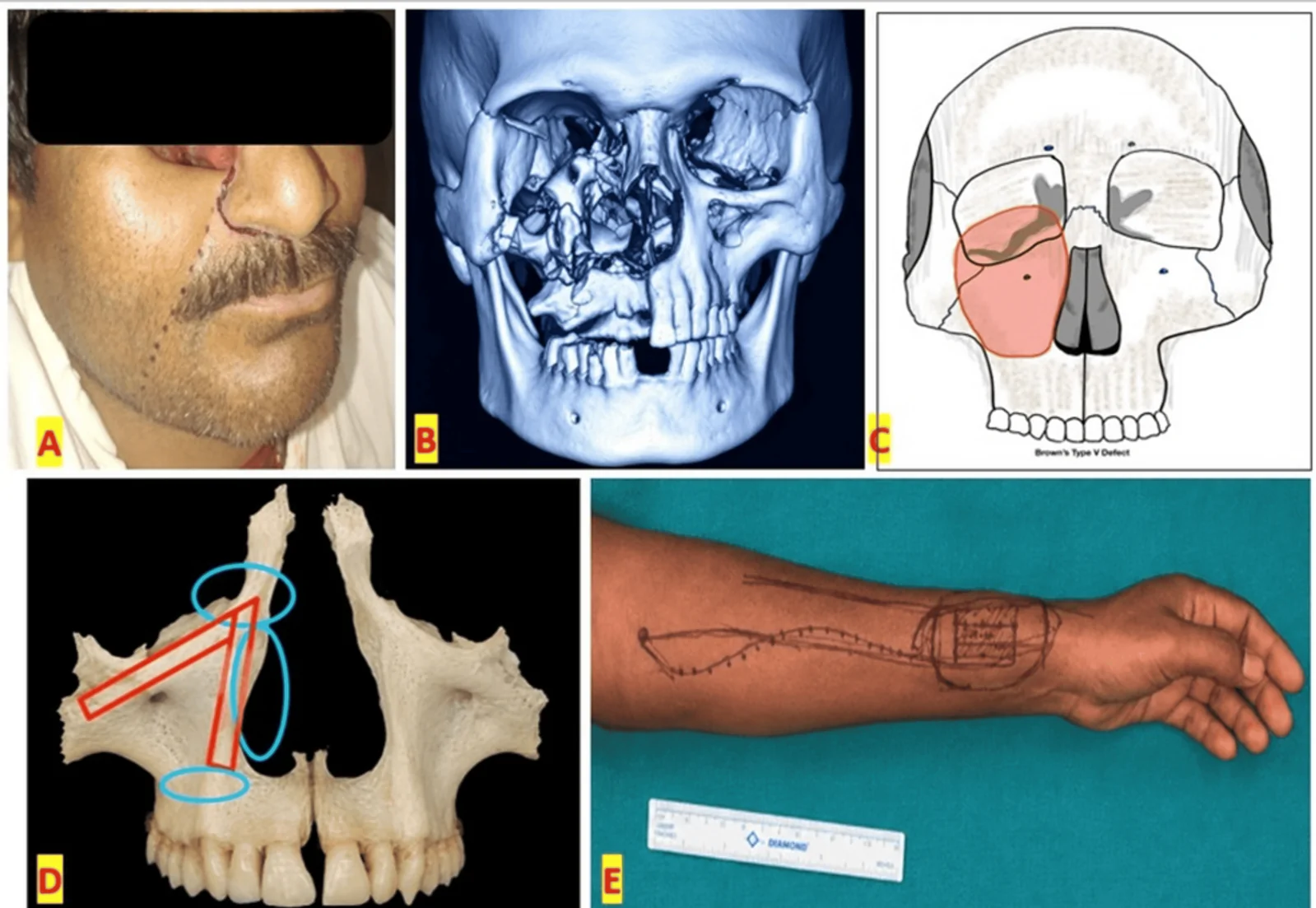
Patient 2. (A) A 44-year-old male with a post-exenteration defect. (B) Non-contrast computed tomography (NCCT) with 3D reconstruction showing a right orbitomaxillary defect, sparing the palate. (C) Illustration showing the defect as per revised Brown classification – type V (Digital drawing created by the corresponding author using Notability). (D) Surgical planning for reconstruction. Red solid lines depict reconstruction of the infraorbital rim and nasomaxillary buttress. Blue solid lines show the placement of the skin flap as lining for the nasal and orbital cavities. (E) Reconstruction plan – Planning of radial forearm free osteocutaneous flap.

**Figure 4:**
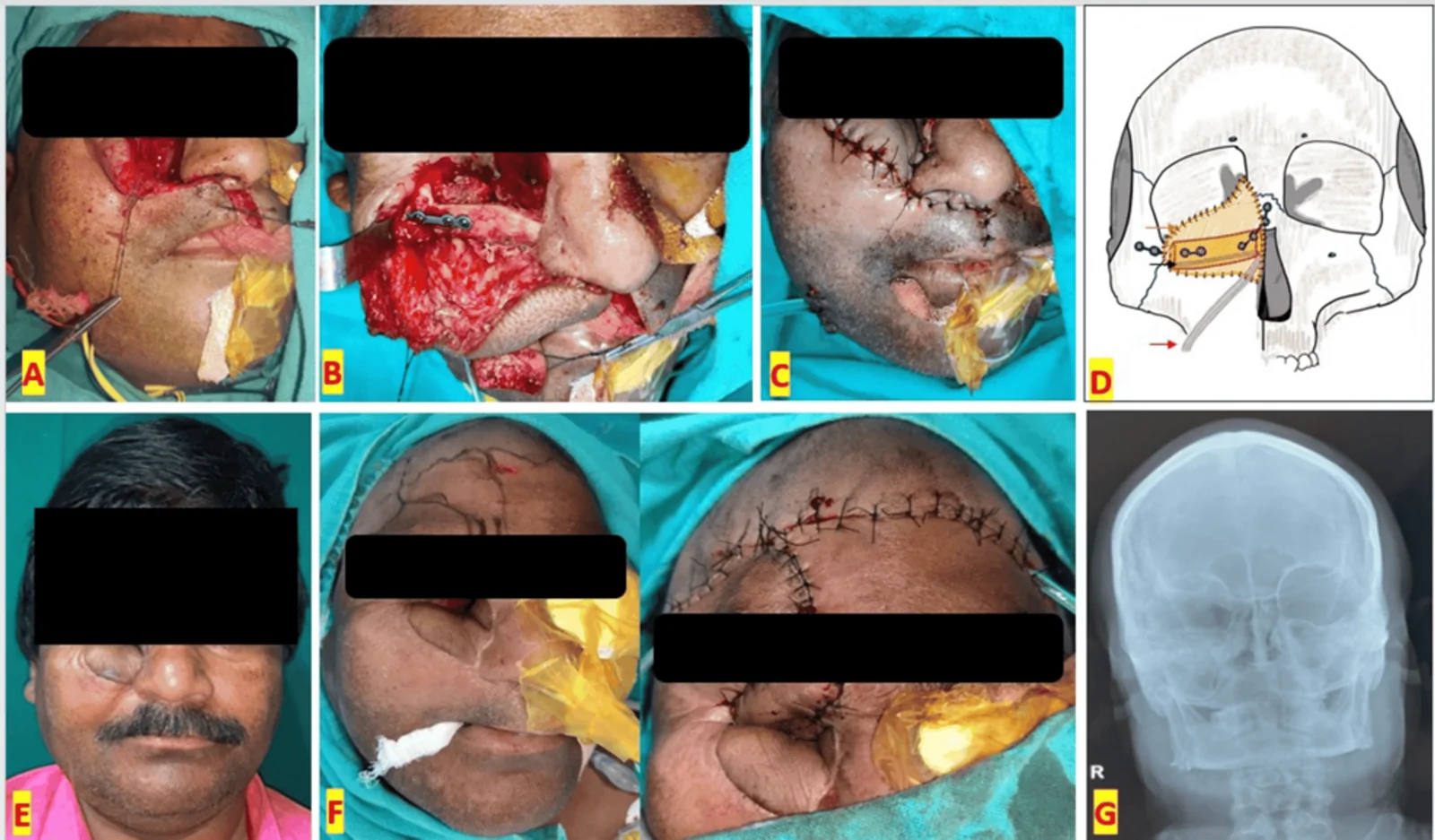
Patient 2. (A) Surgical approach – Weber Ferguson’s approach for the defect and retromandibular approach for donor vessel dissection. (B) Restoration of infraorbital support – Radius fixed to zygoma laterally and nasal bone medially using a 2 mm reconstruction plate. (C) Final closure. (D) Illustration showing planned flap inset (orange arrow – skin paddle as outer skin cover; black arrow – bone fixation; red arrow – pedicle orientation to right side of the neck) (Digital drawing created by the corresponding author using Notability). (E) At four-week follow-up – Well-settled flap, with restoration of inferior orbital rim. (F) Secondary surgery for residual defect over superomedial wall of orbit, resurfaced with ipsilateral paramedian forehead flap. (G) X-ray (posteroanterior view) showing restoration of the infraorbital rim and nasomaxillary buttress using radial forearm osteocutaneous free flap fixed with a 2 mm reconstruction plate.

A 44-year-old male with poorly controlled diabetes mellitus developed mucormycosis during the COVID-19 pandemic, necessitating right orbital exenteration and extensive debridement of the right hemimaxilla. The resulting defect involved the maxillary wall, infraorbital rim, and medial orbital wall, while the palate remained intact. Considering the patient’s comorbid status and predominant soft tissue requirements, reconstruction using a radial forearm osteocutaneous flap was planned. A 6 cm segment of radius, along with a skin paddle, was harvested. The flap was utilized to restore internal lining, provide partial bony support, and obliterate the orbital cavity in preparation for future facial prosthetic rehabilitation. Microvascular anastomosis was performed using the superior thyroid artery and external jugular vein. Free flap monitoring was done hourly on postoperative days 0 and 1, followed by four-hourly on days 2 and 3. Postoperatively, the patient demonstrated satisfactory recovery. Residual superomedial orbital defects were subsequently corrected with an ipsilateral paramedian forehead flap. Subjective improvement in speech and swallowing function was observed at six-month follow-up, and partial restoration of facial symmetry was achieved (Figures 3, 4).

Case 3: Brown type V defect

**Figure 5:**
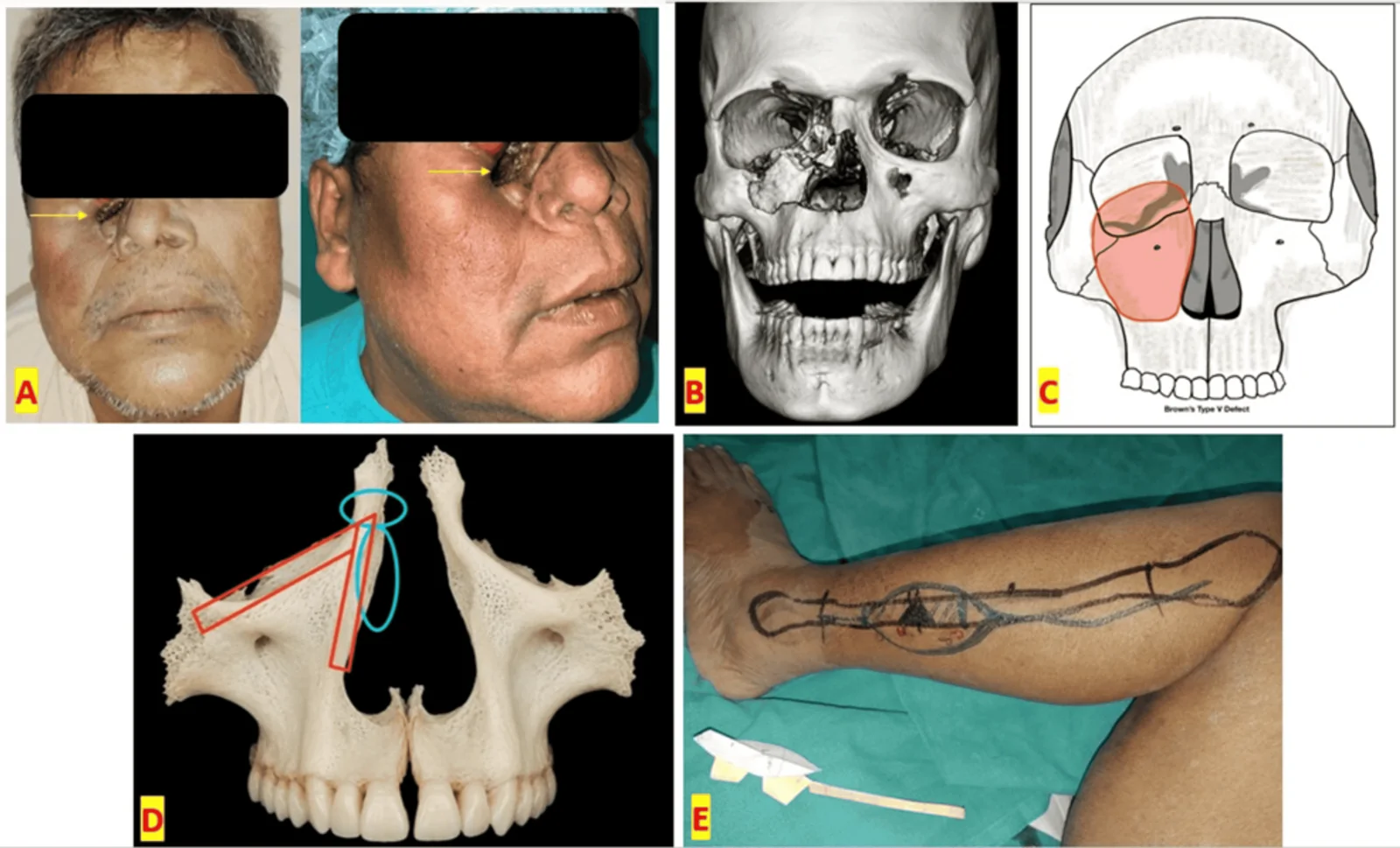
Patient 3. (A) A 51-year-old male with a post-oncological resection defect (yellow arrow) and a failed paramedian forehead flap. (B) Non-contrast computed tomography (NCCT) with 3D reconstruction showing a right orbitomaxillary defect, with the palate intact. (C) Illustration showing the defect as per the revised Brown classification – type V (Digital drawing created by the corresponding author using Notability). (D) Surgical planning for reconstruction. Red solid lines depict reconstruction of the infraorbital rim and nasomaxillary buttress. Blue solid lines show the placement of the skin flap as lining for the nasal and orbital cavities. (E) Reconstruction plan – Planning of fibula osteocutaneous free flap, with a single vertical osteotomy and skin flap for lining.

**Figure 6:**
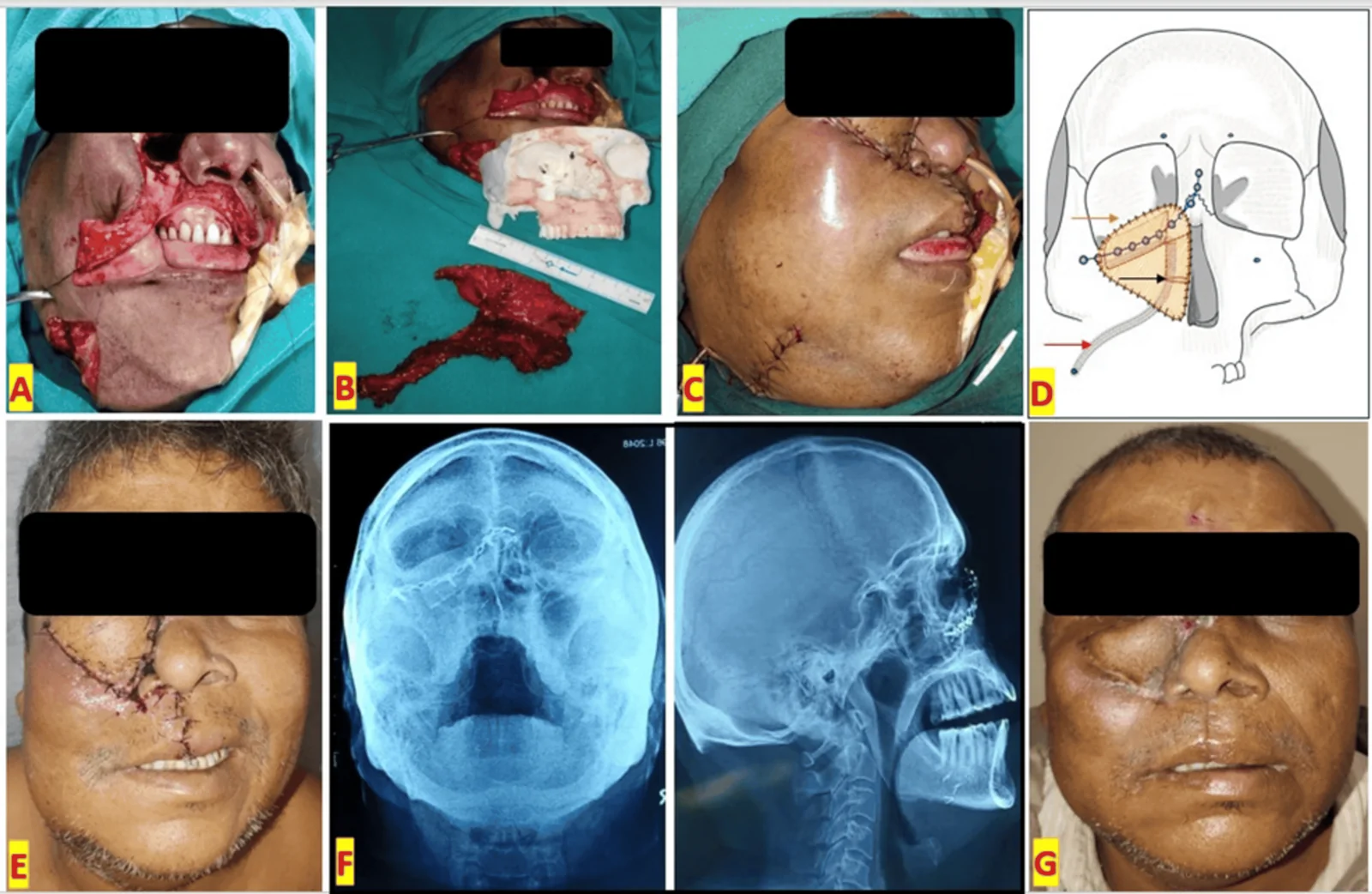
Patient 3. (A) Surgical approach – Weber Ferguson’s approach for the defect and retromandibular approach for donor vessel dissection. (B) 3D model showing the defect with the flap lying in anatomical position. (C) Final closure. (D) Illustration showing planned flap inset (orange arrow – skin paddle as outer skin cover; black arrow – bone fixation; red arrow – pedicle orientation to right side of the neck) (Digital drawing created by the corresponding author using Notability). (E) Immediate postoperative period. Flap healthy with minimal surrounding erythema and edema. (F) X-ray (Waters' view and lateral view) showing restoration of the infraorbital rim and nasomaxillary buttress using a single 2 mm reconstruction plate. (G) At four-week follow-up – Flap settled well.

A 51-year-old male with a history of adenoid cystic carcinoma of the right maxilla, previously operated at another institution two years earlier, presented with failure of a prior paramedian forehead flap reconstruction. Computed tomography imaging demonstrated loss of the infraorbital rim, medial orbital wall, and nasomaxillary buttress with preservation of the palate. Reconstruction using a fibula osteocutaneous free flap was planned. A 9 cm fibular segment was harvested and contoured to reconstruct the maxillary framework, while the skin paddle was utilized for inferomedial orbital lining. Microvascular anastomosis was performed using the superior thyroid vessels. Free flap monitoring was done hourly on postoperative days 0 and 1, followed by four-hourly monitoring on days 2 and 3. The postoperative period was uneventful. At six months of follow-up, the patient underwent minor revision of the nasal base and cheek contour with autologous fat grafting, resulting in satisfactory midfacial projection (Figures 5, 6).

Table 1 summarizes the cases discussed above.

**Table 1:** Summary of the three case presentations.

S. No.	Age/Gender	Etiology	Defect as per the revised Brown classification	Flap used	Recipient vessels	Complications	Secondary procedures	Follow-up outcome
1	35/M	Post-oncologic defect for right maxillary squamous cell carcinoma	Type IIIb	Free fibula osteocutaneous flap	Facial artery, facial vein	Marginal flap necrosis	Debridement with contralateral paramedian forehead flap; intraoral flap debulking at 3 months	Satisfactory speech, swallowing, and orbital position, with acceptable facial contour
2	44/M	Post-COVID mucormycosis with orbital exenteration	Type V	Radial forearm osteocutaneous flap	Superior thyroid artery, external jugular vein	-	Ipsilateral paramedian forehead flap for residual superomedial orbital defect	Satisfactory speech and swallowing with partial restoration of facial symmetry
3	51/M	Post-oncologic defect for right maxillary adenoid cystic carcinoma	Type V	Free fibula osteocutaneous flap	Superior thyroid artery, superior thyroid vein	-	Autologous fat grafting	Satisfactory midfacial projection

## Discussion

Maxillary reconstruction remains a cornerstone of complex midfacial rehabilitation. The reconstructive approach must address the three-dimensional complexity of the defect while restoring not only skeletal continuity but also soft-tissue volume, mucosal lining, and external facial coverage. Functional rehabilitation involving speech, mastication, swallowing, orbital support, and separation of the oral and nasal cavities is equally important in achieving satisfactory long-term outcomes. In the present series, all three patients presented with extensive composite defects, for which prosthetic obturation alone would likely have been inadequate due to poor retention, limited stability, compromised hygiene maintenance, and an inability to adequately restore facial contour and structural support.

The fibula osteocutaneous free flap remains one of the most reliable and versatile options for reconstruction of extensive maxillary defects due to its robust vascularity, long pedicle, ease of harvest, and ability to provide both vascularized bone and soft tissue. In our first and third cases, the fibula flap facilitated accurate reconstruction of the maxillary buttresses and orbital support while also restoring palatal continuity and midfacial contour. Its dense cortical bone permits multiple osteotomies without compromising vascularity, thereby allowing recreation of the complex three-dimensional maxillary framework. Furthermore, the quality and quantity of fibular bone make it particularly suitable for future osseointegrated dental rehabilitation. The associated skin paddle also provides a reliable intraoral lining or external soft-tissue resurfacing when required [2,6].

In the second case, reconstruction was performed using a radial forearm osteocutaneous free flap because the patient had significant systemic comorbidities and comparatively lower structural bony requirements. Although this flap provides limited bone stock when compared with the fibula, it offers a thin, pliable, and well-vascularized skin paddle that is highly suitable for internal lining, cavity obliteration, and soft tissue resurfacing. When harvested as an osteocutaneous flap, it can provide moderate skeletal support to the infraorbital and malar regions while maintaining low donor-site morbidity. Additional advantages include reliable vascular anatomy, relative ease of harvest, shorter operative time, and suitability for elderly or medically compromised patients [8]. These findings are consistent with previous studies supporting the use of the radial forearm osteocutaneous flap in small-to-moderate composite midfacial defects [3,7].

The application of revised Brown’s classification system proved particularly valuable for our surgical planning and decision-making. Beyond simple categorization of defects, the classification assists in anticipating reconstructive complexity, selecting the most appropriate flap, and predicting functional and aesthetic outcomes [5]. Type IIIb defects involving the orbital floor require meticulous restoration of orbital support to prevent complications such as enophthalmos, diplopia, and orbital dystopia. In contrast, type V defects are associated with more extensive three-dimensional tissue loss and therefore benefit significantly from the versatility of osteocutaneous free flaps, particularly the fibula flap.

In our series, fibula flaps were preferred when restoration of maxillary buttresses and orbital support required substantial vascularized bone, whereas the radial forearm osteocutaneous flap was selected when soft tissue requirements predominated and structural demands were limited. Donor-site morbidity was acceptable in all three patients. No significant donor-site complications were encountered during follow-up, supporting the continued use of fibula and radial forearm osteocutaneous flaps as reliable reconstructive options when selected according to defect characteristics and patient factors.

Several alternative reconstructive options, including subscapular system flaps and the deep circumflex iliac artery (DCIA) flap, have been described for complex maxillary defects. These flaps provide varying combinations of vascularized bone and soft tissue, allowing reconstruction to be tailored to defect characteristics [1]. Ultimately, flap selection depends on tissue requirements, patient factors, donor-site considerations, and surgeon expertise.

Recent advances in virtual surgical planning and three-dimensional printing have enhanced the precision of maxillary reconstruction by improving osteotomy planning, facial symmetry, and occlusal restoration [9]. Although not applied in our series, these technologies have the potential to further optimize functional and aesthetic outcomes.

Postoperative recovery in all three patients was satisfactory, with no instances of total flap loss or major vascular compromise, underscoring the reliability and safety of microvascular reconstruction in experienced hands. Minor secondary procedures, including flap thinning, contour refinement, and soft tissue revision, were required in selected cases to optimize aesthetic outcomes. This highlights the importance of staged reconstruction and long-term follow-up in achieving comprehensive facial rehabilitation [10].

Despite having a small number, heterogeneous pathology, and limited follow-up, this series highlights the importance of classification-guided, individualized reconstructive planning in the management of complex maxillary defects. Microvascular osteocutaneous free flaps provide a reliable single-stage solution for restoring midfacial support, oral function, and facial aesthetics following oncologic resection and invasive infections such as mucormycosis. Larger multicentric studies with longer follow-up are needed to evaluate long-term outcomes, establish standardized reconstructive protocols, and assess the potential role of emerging technologies such as virtual surgical planning in improving surgical precision and reproducibility.

## Conclusions

Autologous microvascular osteocutaneous free flaps provide a feasible reconstructive option for the management of complex maxillary defects, as demonstrated by this case series. In our experience, individualized flap selection guided by the revised Brown classification facilitated reconstructive planning and restoration of form and function. Given the limited sample size and heterogeneity of cases, these findings should be interpreted as illustrative, and larger studies are warranted to further evaluate long-term outcomes and refine reconstructive decision-making.
